# Supplementary material for: NeuroCarta: An automated and quantitative approach to mapping cellular networks in the mouse brain
Source: Netw Neurosci. 2025 Oct 30;9(4):1279–98. doi: 10.1162/NETN.a.33 (PMC12594485; doi:10.1162/NETN.a.33)
Supplement: Supplementary file 1 [file netn-9-4-1279-s001.pdf]

Supplemental figures and tables

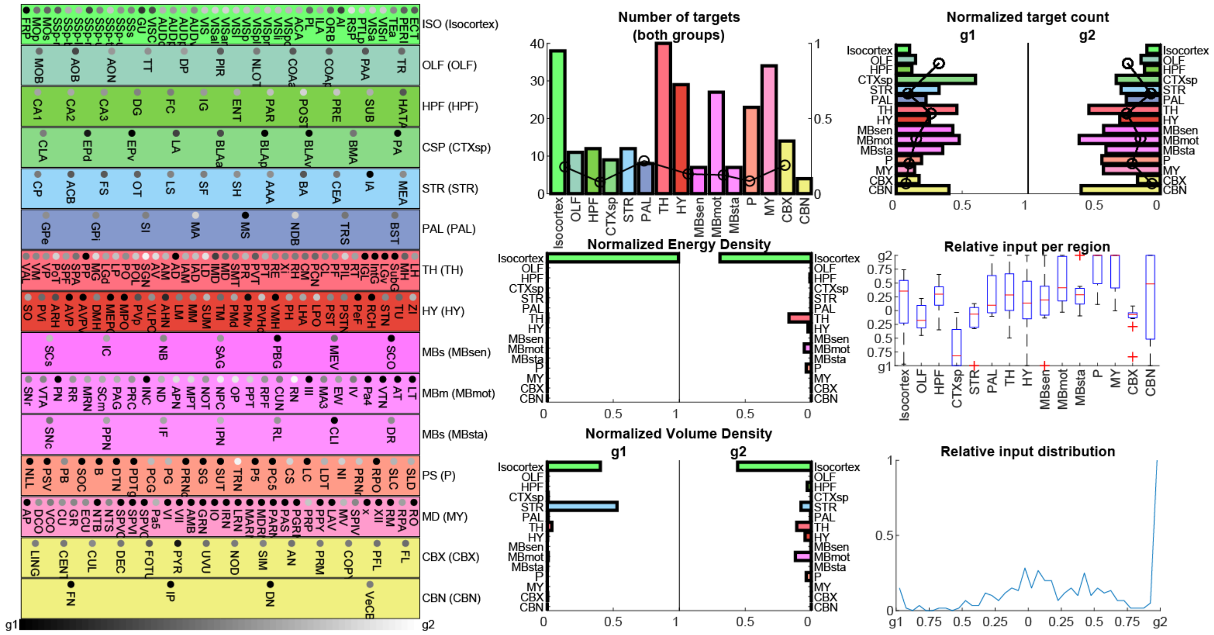

Supplemental figure 1. Example of the function output of the *crosscorrelatemaps* function. The *crosscorrelatemaps* function generates a MATLAB figure showing statistical differences between two experiments. Specifically, this example compares the experiments with ids 100140756 (indicated in the figure as g1) and 100140949 (indicated as g2). The figure on the left shows the relative projection density of projections towards postsynaptic targets, originating in the experiments' respective projection sites. The smaller figures on the right show various statistical distributions comparing the two experiments, i.e. the total number of postsynaptic targets in the two experiments combined; the normalized number of targets, projection density, and projection volume per larger brain area shown separately for the two experiments; and the distributions of incoming projections ratios between the two experiments.

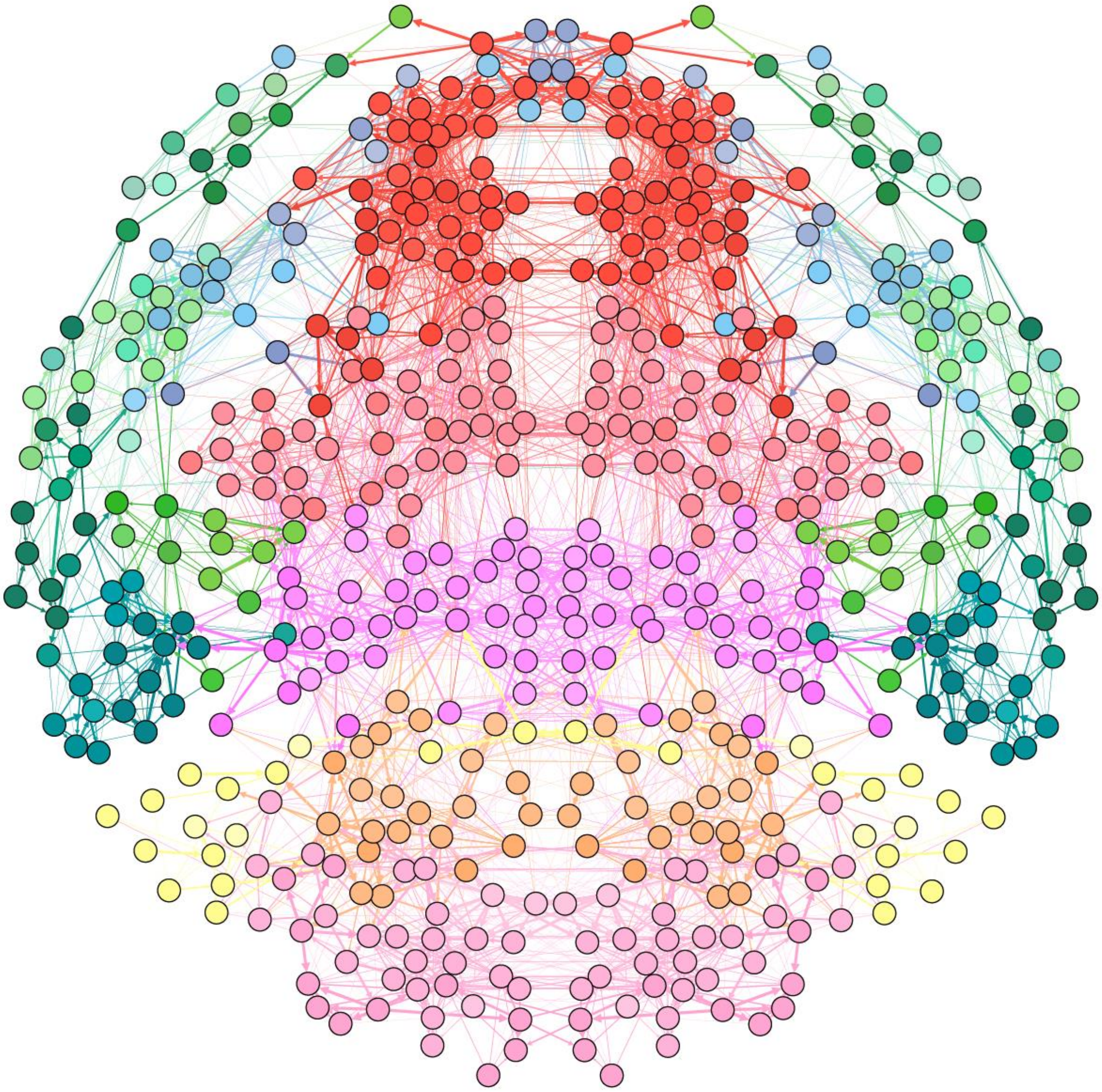

Supplemental figure 2. Bilateral mouse brain visualized in Gephi. The density-based mouse brain network constructed by NeuroCarta was exported in GEXF file format and then visualized in Gephi.

Using the Fruchterman-Reingold algorithm, a network layout was generated based solely on the network connectivity. For visibility reasons, only 10% of the strongest edges are shown. Node coloring is the same as used in the AMBCA (green = cortex, red = interbrain, purple = midbrain and hindbrain, yellow = cerebellum).

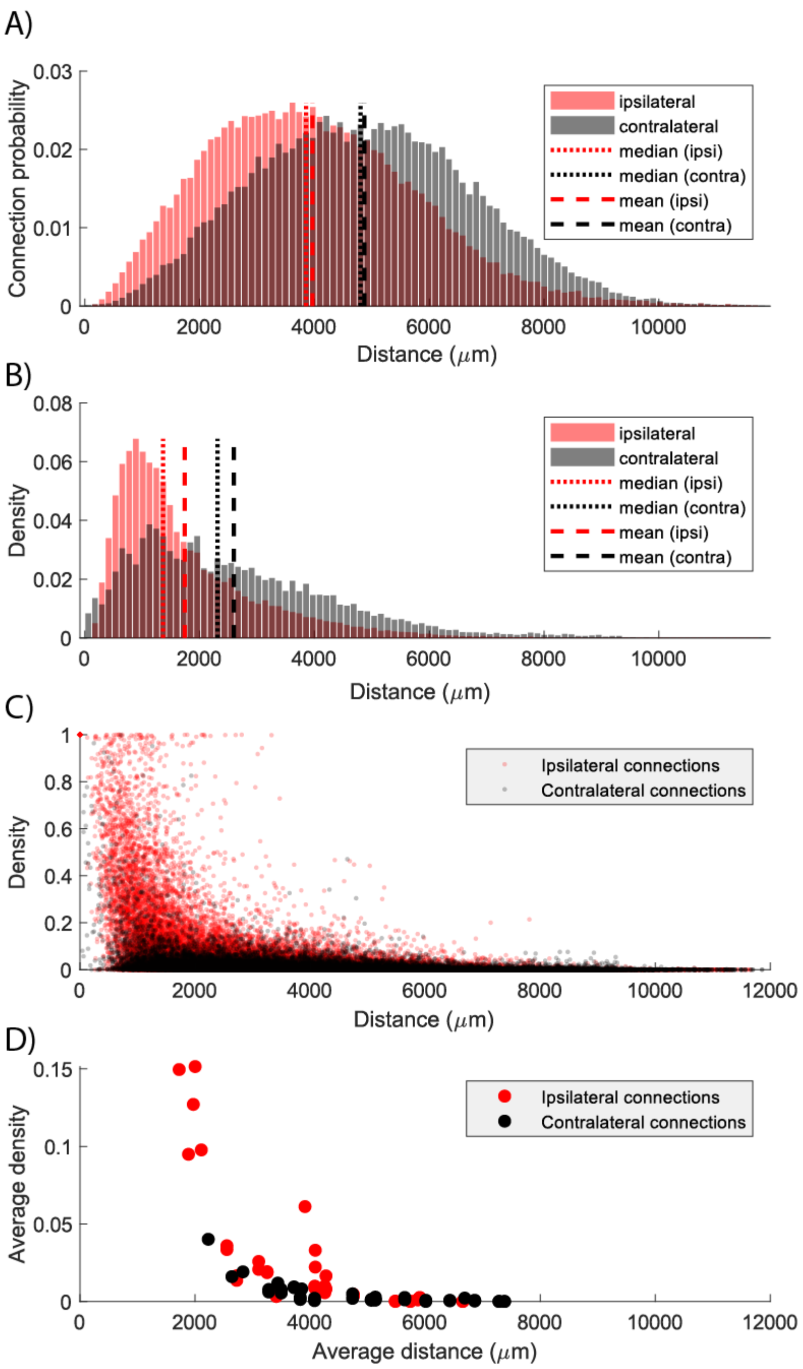

Supplemental figure 3. Projection density declines over growing euclidean distance. A) The probability of connection (i.e. number of connections normalized to the area under curve) as a function of Euclidean distance between the presynaptic source of the projection and postsynaptic target, shown separately for ipsi- and contralateral projections. Distances were calculated from the geometric centers

of the targets. **B)** Distribution of projection densities as a function of Euclidean distance. **C)** Projection density plotted against Euclidean distance for all existing ipsi- and contralateral projections. **D)** Projection density plotted against Euclidean distance, averaged per larger brain region.

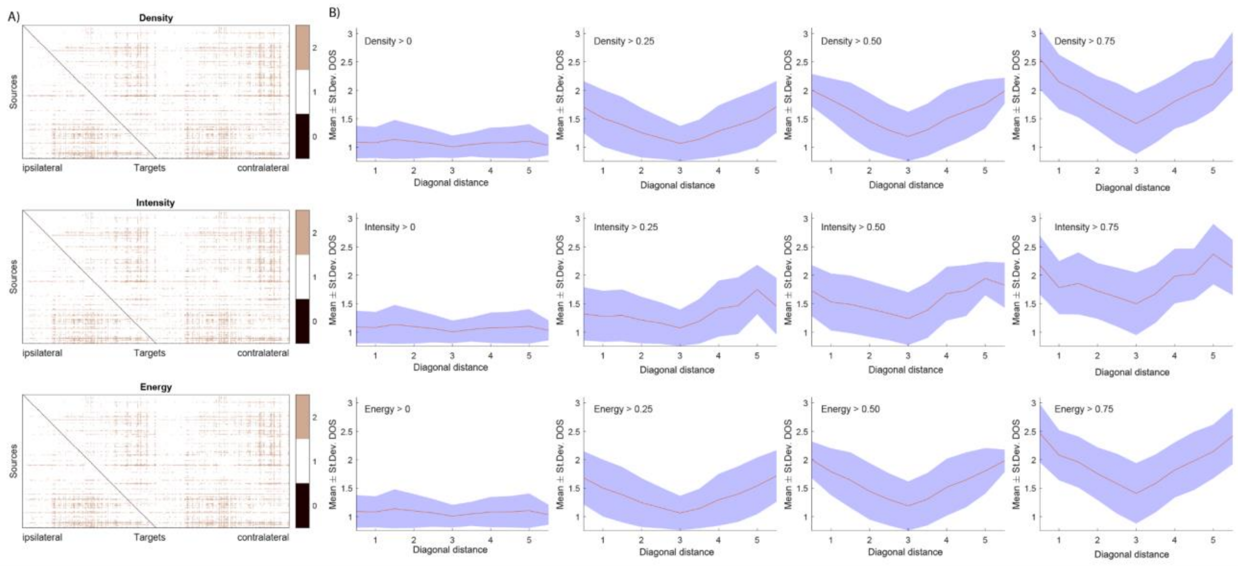

Supplemental figure 4. Degree of separation. A) The degree of separation (DOS) matrices for an non-thresholded bilateral, density-based shown for networks based on projection density, intensity and energy. **B)** The average DOS across the positive diagonals of the respective averaged DOS matrices from Figure 4A, showing that DOS is generally lower within the larger brain regions.

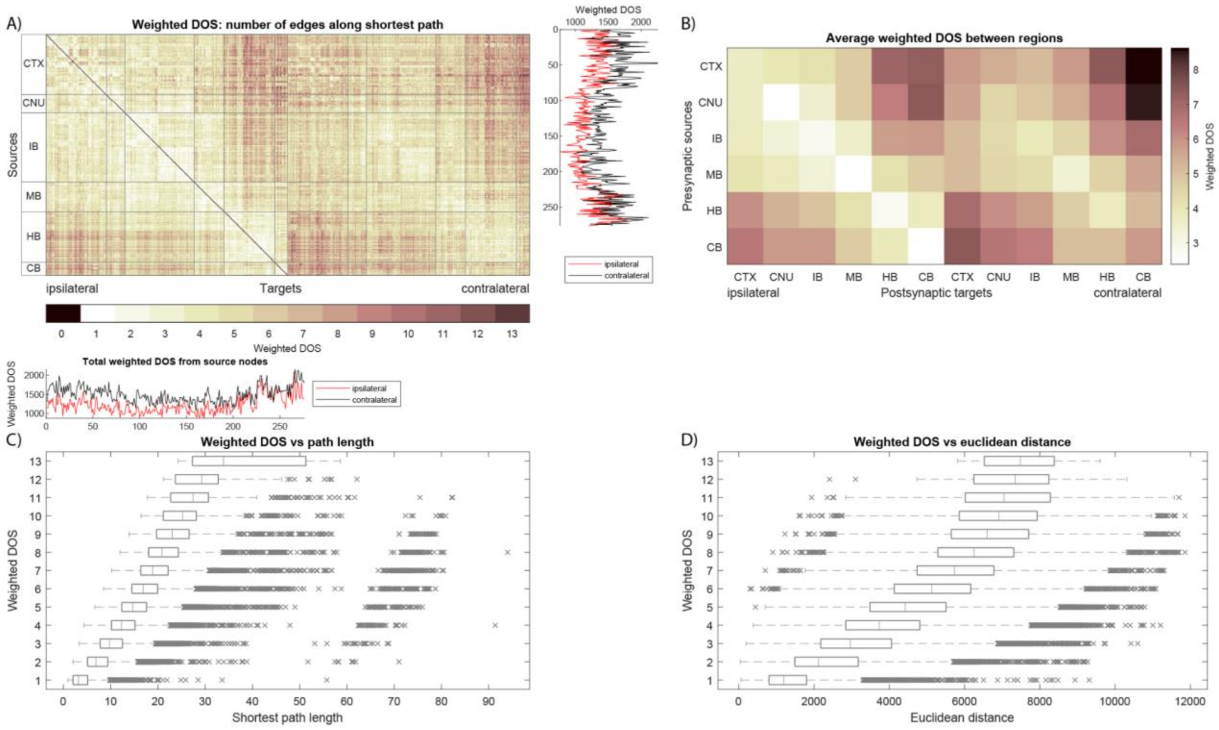

Supplemental figure 5. Weighted degree of separation. A) Weighted degree of separation (weighted DOS) between any pair of nodes in the bilateral, density-based network. Row-wise sums (for outgoing weighted DOS) and column-wise sums (for incoming weighted DOS) are shown in the line plots on the sides. **B)** Weighted DOS averaged within larger brain areas. **C)** Distribution of weighted distances for each occurring value of weighted DOS, showing the relation between the two. **D)** Distribution of euclidean distances for each occurring value of weighted DOS.

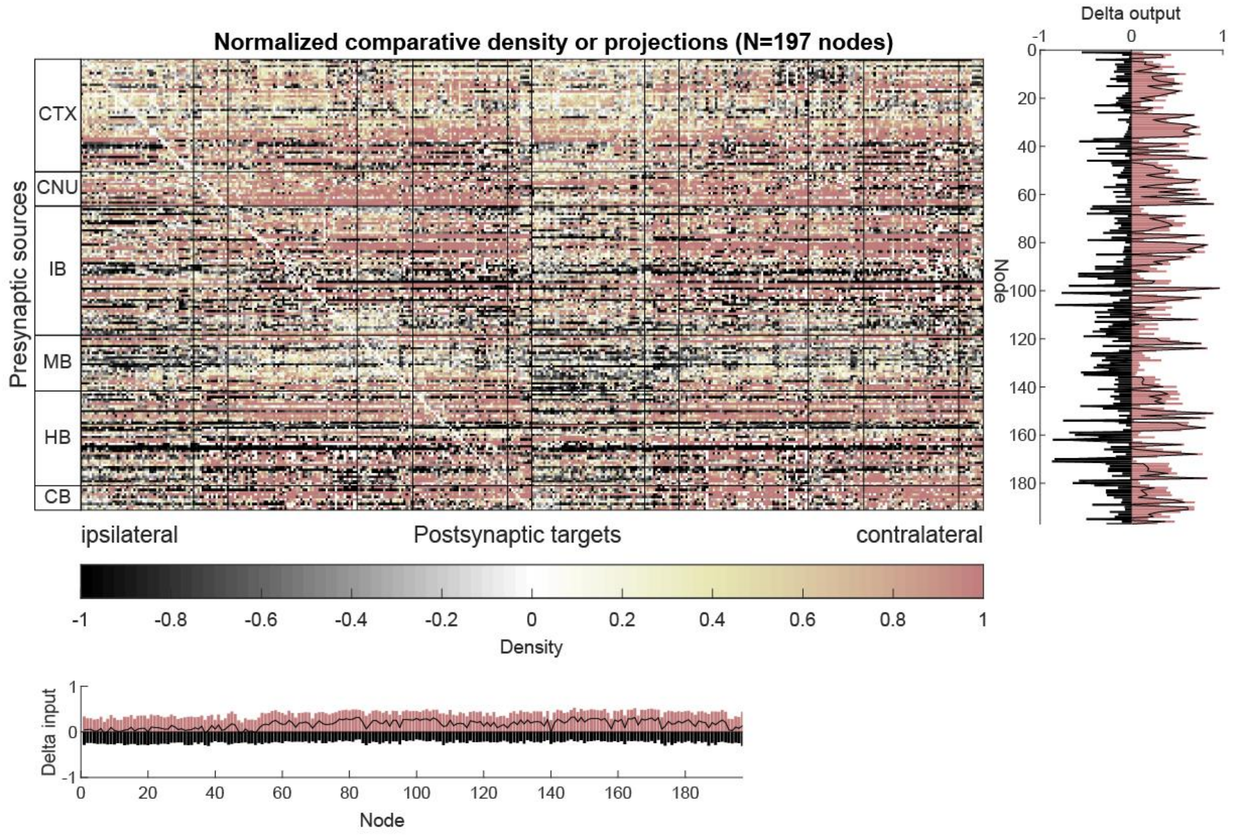

Supplemental figure 6. Relative density matrix to compare connectivity between sexes. Relative density shown for all nodes in the bilateral network. A value of -1 means a projection only exists in female mice, a value of 1 means a projection only exists in male mice, a value of 0 means the connection has equal strength in both sexes, and any value in between indicates a connection that is relatively stronger in one of the sexes. The area plots on the sides show the row-wise and column-wise sums of the matrix, indicating which brain areas either receive more incoming projections in a particular sex, or send more outgoing projections.

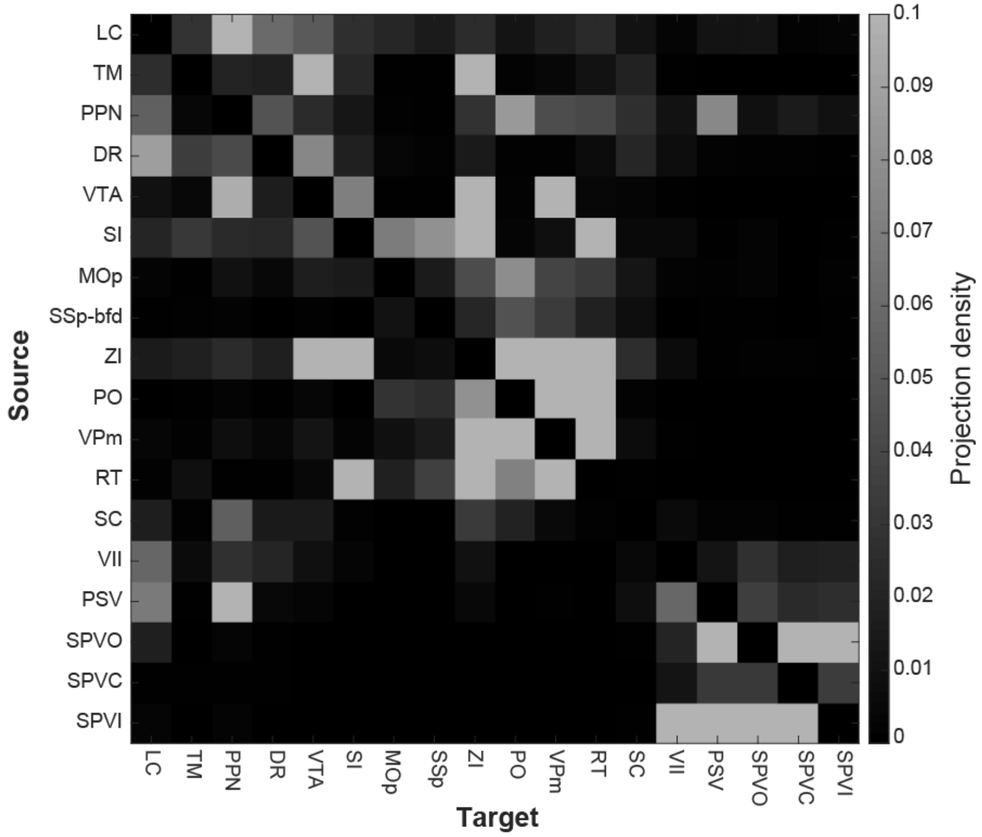

Supplemental figure 7. Connectivity matrix of the whisker system. Density-based connectivity matrix of the whisker system using the nodes selected by Raoult et al. (Raoult et al. 2024). This matrix contains the same data as the one in Figure 7A except for omitting the sex-specific projections.

Supplemental table 1: Node acronyms

Acronym	Brain structure name (taken from AMBCA)
ACVII	Accessory facial motor nucleus
AD	Anterodorsal nucleus
AI	Agranular insular area
AMB	Nucleus ambiguus
AUDd	Dorsal auditory area
BAC	Bed nucleus of the anterior commissure
CB	Cerebellum
CBN	Cerebellar nuclei
CBX	Cerebellar cortex
cDG	Dentate gyrus (contralateral to source node)
CM	Central medial nucleus of the thalamus
CNU	Cerebral nuclei
COPY	Copula pyramidis
CP	Caudoputamen

CS	Superior nucleus raphe
CTX	Cerebral cortex
CTXsp	Cortical subplate
DCO	Dorsal cochlear nucleus
DG	Dentate gyrus
DR	Dorsal nucleus raphe
EW	Edinger-Westphal nucleus
FRP	Frontal pole
HB	Hindbrain
HPF	Hippocampal formation
HY	Hypothalamus
IB	Interbrain
IF	Interfascicular nucleus raphe
ILA	Infralimbic area
IMD	Interomedial dorsal nucleus of the thalamus
IO	Inferior olivary complex
LA	Lateral amygdalar nucleus

LC	Locus coeruleus
LDT	Laterodorsal tegmental nucleus
LING	Lingula
LM	Lateral mammillary nucleus
LPO	Lateral preoptic area
MA	Magnocellular nucleus
MA3	Medial accessory oculomotor nucleus
MB	Midbrain
MBmot	Midbrain, motor related
MBsen	Midbrain, sensory related
MBsta	Midbrain, behavioral state related
MEPO	Median preoptic nucleus
MOp	Primary motor area
MRN	Midbrain reticular nucleus
MS	Medial septal nucleus
MY	Medulla
NDB	Diagonal band nucleus

NLL	Nucleus of the lateral lemniscus
NLOT	Nucleus of the lateral olfactory tract
OLF	Olfactory areas
ORB	Orbital area
P	Pons
P5	Peritrigeminal zone
PAG	Periaqueductal gray
PAL	Pallidum
PERI	Perirhinal area
PL	Prelimbic area
PMd	Dorsal premammillary nucleus
PN	Paranigral nucleus
PO	Posterior complex of the thalamus
PP	Peripeduncular nucleus
PPN	Pedunculopontine nucleus
PRNc	Pontine reticular nucleus, caudal part
PSV	Principal sensory nucleus of the trigeminal

PVi	Periventricular hypothalamic nucleus, intermediate part
PVT	Paraventricular nucleus of the thalamus
RH	Rhomboid nucleus
RO	Nucleus raphe obscurus
RPO	Nucleus raphe pontis
RSP	Retrosplenial area
RT	Reticular nucleus of the thalamus
SAG	Nucleus sagulum
SC	Superior colliculus
SFO	Subfornical organ
SI	Substantia innominata
SPA	Subparafascicular area
SPVC	Spinal nucleus of the trigeminal, caudal part
SPVI	Spinal nucleus of the trigeminal, interpolar part
SPVO	Spinal nucleus of the trigeminal, oral part
SSp-bfd	Primary somatosensory area, barrel field
SSp-tr	Primary somatosensory area, trunk

STR	Striatum
TH	Thalamus
TM	Tuberomammillary nucleus
TRS	Triangular nucleus of septum
VII	Facial motor nucleus
VIS	Visual areas
VISal	Anterolateral visual area
VISC	Visceral area
VISp	Primary visual area
VPM	Ventral posteromedial nucleus of the thalamus
VTA	Ventral tegmental area
Xi	Xiphoid thalamic nucleus
ZI	Zona incerta

Supplemental note 1: Example code

```
%% Download and import the AMBCA data

% This process takes a while but needs to be ran only once.

build_database;



%% Top 20 areas targeted by Barrel cortex

% Load metadata

load('experiments', 'experiments');

load('nodelist', 'nodelist');



% Load experiments targeting barrel cortex and average them

barrel_experiments = contains(experiments.injection_site, 'barrel field');

experiment_ids = experiments.id(barrel_experiments);

data = groupexperiments(experiment_ids);



% Display top 20 most strongly targeted areas from barrel cortex

[~, index] = maxk(data.ipsi', 21);

disp('Top 20 areas targeted by Barrel cortex:');

disp(nodelist(index(2:end)));
```

```matlab
%% Wildtype network

% Select experiments in wildtype mice

wildtype_exp = findexperiments('strain', 'C57BL/6J', 'transgenic_line',
'""');

% Construct the network

network = loadmap('density', 'density', wildtype_exp);

% Create a condensed network

macro_network = macromap(network);



% Create the figure

load('structures', 'groups');

figure;

imagesc(macro_network);

axis image;

set(gca, 'xtick', 1:30, 'xticklabels', [groups.name groups.name]);

set(gca, 'ytick', 1:15, 'yticklabels', groups.name);

title('Average projection density in wildtype mice');

xlabel('Target');

ylabel('Source');
```

```matlab
%% Sex comparison

% Group experiments by mouse sex and create two separate networks

male_experiments = findexperiments('gender', 'M');

female_experiments = findexperiments('gender', 'F');

male_network = loadmap('density', 'density', male_experiments);

female_network = loadmap('density', 'density', female_experiments);

male_macro = macromap(male_network);

female_macro = macromap(female_network);

difference = (male_macro - female_macro) ./ (male_macro + female_macro);


% Create the figure

load('structures', 'groups');

figure;

subplot(3, 1, 1); imagesc(male_macro); axis image; colorbar; title('Male');

set(gca, 'xtick', 1:30, 'xticklabels', [groups.name groups.name]);

set(gca, 'ytick', 1:15, 'yticklabels', groups.name);

subplot(3, 1, 2); imagesc(female_macro); axis image; colorbar;
title('Female');

set(gca, 'xtick', 1:30, 'xticklabels', [groups.name groups.name]);
```

```
set(gca, 'ytick', 1:15, 'yticklabels', groups.name);

subplot(3, 1, 3); imagesc(difference); axis image; colorbar;
title('Relative');

set(gca, 'xtick', 1:30, 'xticklabels', [groups.name groups.name]);

set(gca, 'ytick', 1:15, 'yticklabels', groups.name);



%% Finding hubs in wildtype mice

% Select experiments in wildtype mice

wildtype_exp = findexperiments('strain', 'C57BL/6J', 'transgenic_line',
'""');

% Construct the network

network = loadmap('density', 'density', wildtype_exp);

% Create a condensed network

macro_network = macromap(network);

% Compute shortest paths in the network

[~, paths] = getpaths(network);

% Compute betweenness centralities

centrality = getcentrality(paths);



% Create the figure

load('nodelist', 'ids');
```

```
load('structures', 'structures', 'regions', 'groups');

[~, index] = ismember(ids, structures.id);

figure;

reg = structures.region(index)';

subplot(3, 1, 1); imagesc(network); axis image; colorbar;

subplot(3, 1, 2); imagesc(macro_network); axis image; colorbar;

set(gca, 'xtick', 1:30, 'xticklabels', [groups.name groups.name]);

set(gca, 'ytick', 1:15, 'yticklabels', groups.name);

subplot(3, 1, 3); boxplot(centrality, [reg reg]);

set(gca, 'xticklabels', groups.name);

ylabel('Betweenness centrality');
```
